# Herpes Simplex Proctitis Mimicking Inflammatory Bowel Disease in a Teenaged Male

**DOI:** 10.1155/2017/3547230

**Published:** 2017-04-04

**Authors:** Kristen E. Sandgren, Nathan B. Price, Warren P. Bishop, Patrick J. McCarthy

**Affiliations:** Stead Family Department of Pediatrics, Roy J. and Lucille A. Carver College of Medicine, University of Iowa, Iowa City, IA, USA

## Abstract

We report the case of a 17-year-old male who was initially assessed for pain with defecation, bloody rectal discharge, and diarrhea, consistent with proctitis. Though proctitis is most commonly due to inflammatory bowel disease (IBD), infectious etiologies must also be considered, including sexually transmitted causes of infectious proctitis. In discussion of his sexual history, he identified as homosexual and acknowledged engaging in receptive anal intercourse. Rectal biopsies obtained via colonoscopy were culture-positive for herpes simplex virus (HSV), leading to a diagnosis of HSV proctitis and treatment with an appropriate antiviral medication. HSV proctitis is more common in individuals with high-risk sexual practices, including men who have sex with men. While this may be an uncommon diagnosis for pediatricians to make in practice, a high clinical index of suspicion for sexually transmitted infectious proctitis in those with risk factors must be maintained in order to facilitate appropriate testing, treatment, and counseling of affected individuals.

## 1. Case Presentation

A 17-year-old male was admitted to the hospital after presenting with 6 days of rectal bleeding, diarrhea, purulent rectal drainage, and tenesmus. He complained of bloody and mucoid stools. He did not have rectal bleeding prior to this illness. He reported no skin lesions of his genital or anal areas. He had not been eating or drinking well during the course of this illness due to lack of appetite. He denied any abdominal pain, fevers, night sweats, oral lesions, or arthralgia. On the evening prior to admission, he developed an erythematous, pruritic, slightly raised rash without vesicles or central clearing on his trunk that spread to his buttocks and legs. He described intermittent blurry vision that started at about the same time as his stool changes, without any eye pain.

Past medical history included one episode of candidal esophagitis that was successfully treated with fluconazole. He was noted to have mild lymphopenia at that time, and for which he underwent an extensive work-up for immunodeficiency, including a normal complete blood count (CBC) with differential, a normal peripheral blood smear, and negative human immunodeficiency virus (HIV) antibodies. Flow cytometry showed minimally decreased absolute numbers of total T-cells, CD4 T-cells, and CD8 T-cells and was deemed unlikely to result in increased susceptibility to infections by an immunologist.

Family history was significant for ulcerative colitis in his maternal grandmother. Social history was significant for oral and receptive anal intercourse on multiple occasions, with last sexual encounter within the past month. Condom use was inconsistent. He denied knowledge of his partner having any sexually transmitted infections. He denied personal history of sexually transmitted infections or sexual abuse.

Upon admission, physical exam showed an alert and cooperative teenaged male with vital signs remarkable for mild tachycardia. Skin exam showed a pruritic morbilliform rash on the neck, upper extremities, trunk, buttocks, and lower extremities with dermatographism. Petechiae were noted on the palate. External rectal exam was performed and showed tenderness and erythema around the perianal region as well as a single anal fissure at the 2 o'clock position. Genitourinary exam showed Tanner V genitalia without urethral discharge. No perianal or genitourinary cutaneous lesions were present. Cardiopulmonary, abdominal, neurological, and funduscopic examinations were all normal.

**Figure 1:**
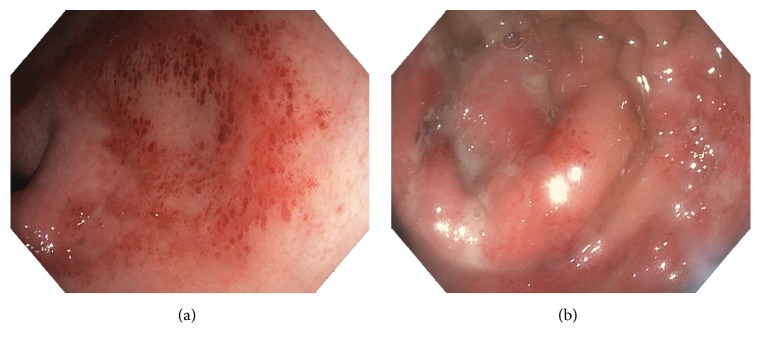
Photos taken of the rectal mucosa during colonoscopy. (a) Erythema and friability of the rectal mucosa are present. Multiple ulcerated lesions are also evident. (b) Additional ulcerated lesions and mucopurulent exudates are present.

Laboratory evaluation included negative results for syphilis IgG, hepatitis C antibody, hepatitis B surface antigen, cytomegalovirus (CMV) IgM, CMV polymerase chain reaction (PCR), HIV antigen and antibody, and HIV RNA PCR from blood;* Neisseria gonorrhoeae* and* Chlamydia trachomatis* PCR from rectal swab and urine; and culture and Shiga toxin from stool. CBC with differential was normal and without previously described lymphopenia. Erythrocyte sedimentation rate (ESR) was normal and C-reactive protein (CRP) was mildly elevated at 1.8 mg/dL (normal value < 0.5 mg/dL). To assess for inflammatory bowel disease (IBD) and other etiologies of symptoms, upper endoscopy and colonoscopy were performed. Upper endoscopy showed normal mucosa with no signs of candidiasis. Colonoscopy showed severe erythema, ulceration, and friability of the rectum with exudate, concerning for a viral process versus IBD (Figure 1). More proximal portions of the colon were not affected. Rectal biopsies were obtained and sent for* N. gonorrhoeae* and* C. trachomatis* PCR, which were negative, and viral culture, which was positive for herpes simplex virus (HSV). Subtyping of the HSV isolate was not conducted. Microscopic examination of the biopsy specimen was without histopathological findings concerning for IBD.

Given his symptoms of blurry vision, ophthalmology was consulted due to concern for HSV keratoconjunctivitis and eye exam was found to be normal without dendritic lesions. Dermatology was consulted to assess the rash, and they diagnosed a morbilliform eruption with dermatographism. This was thought to be triggered by concomitant HSV infection.

He was initially treated with intravenous fluids and pain control. After diagnosis of HSV infection, he was treated with valacyclovir 1 gram (g) orally twice daily for 10 days. His rash improved with antihistamines and topical triamcinolone. His vision complaints improved with rehydration, and vision symptoms were therefore attributed to dehydration. His proctitis symptoms gradually improved, and he was discharged on hospital day 5. Counseling on safe sexual practices was given prior to discharge. On initial follow-up 10 days after discharge, he was asymptomatic with normal stooling pattern and resolution of rash. He has subsequently been lost to follow-up.

## 2. Discussion

Proctitis is an inflammatory process of the lining of the distal colon and rectum [1]. Symptoms of proctitis include anorectal pain, tenesmus, mucopurulent exudates, and hematochezia [2]. Proctitis is most commonly associated with IBD, though other noninfectious and infectious causes are possible [1]. Infectious proctitis must be considered prior to immunosuppressant therapy for presumed IBD, as immunosuppressant medications may result in a lack of improvement or clinical worsening of infectious proctitis [3]. In males who have sex with males (MSM) and others who engage in receptive anal intercourse, sexually transmitted infections must be considered. The most common sexually transmitted causes of proctitis include* N. gonorrhoeae*,* C. trachomatis*,* Treponema pallidum*, and HSV [2]. Other organisms such as CMV,* Entamoeba histolytica*,* Campylobacter* sp., and* Shigella* sp. can cause proctitis as well, especially in HIV infected patients [4]. HSV is second only to* N. gonorrhoeae* as a sexually transmitted cause of infectious proctitis in homosexual males [2]. Patients presenting with acute proctitis should receive anoscopy and be evaluated for infection with testing for HSV,* N. gonorrhoeae*,* C. trachomatis*,* T. pallidum,* and HIV [2, 4]. Individuals with acute proctitis who have recently practiced receptive anal intercourse should be treated with presumptive therapy for* N. gonorrhoeae* and* C. trachomatis*, and individuals with HIV and/or painful perianal or mucosal ulcers should also be treated presumptively for anogenital HSV [4].

In addition to symptoms of infectious proctitis listed above, HSV proctitis can present with constipation, sacral paresthesia, and difficulty urinating, possibly related to autonomic nervous system dysfunction associated with the neurotropic nature of HSV [5]. These symptoms of HSV proctitis usually begin after an incubation period of 2–12 days [1, 6]. HSV proctitis can be caused by HSV-1 or HSV-2, but approximately 70% of cases are caused by HSV-2 [7]. Though HSV proctitis can occur in the setting of primary infection or viral reactivation, most cases are associated with primary infection [1]. Viral reactivation can occur in response to trauma, fever, stress, or ultraviolet radiation [6]. Endoscopic findings are generally in the distal 10–15 centimeters (cm) of the colon and include friable mucosa, diffuse distal ulcerations, and vesicular lesions [1, 5, 7]. Absence of external herpes lesions should not decrease suspicion for HSV proctitis, as only ~30% of men with herpes proctitis have external lesions at the time of diagnosis [8].

MSM and HIV-positive individuals are at higher risk of HSV proctitis. Compared to the seroprevalence of HSV-1 and HSV-2 of 57.7% and 17.0%, respectively, in the general population of the United States [9], the seroprevalence in MSM is significantly higher. 95% of MSM are seropositive for HSV-1, HSV-2, or both, and a 2004 retrospective review showed 16% of MSM with proctitis had HSV detected by culture methods [7, 10]. HSV-1 and HSV-2 are both more common among HIV-positive men than HIV-negative men. HIV-positive individuals have increased symptomatic and asymptomatic shedding of HSV (especially when CD4 counts are low), increasing risk of transmission to sexual partners [11, 12]. HSV also has implications in regard to transmission of HIV. Symptomatic and asymptomatic HSV have been shown to increase HIV shedding, and those with rectal mucosal inflammation and ulceration associated with HSV likely have increased susceptibility to HIV infection due to the nonintact nature of the mucosal barrier [7, 8]. Given the relationship between HSV and HIV, it is important that all patients who present with HSV proctitis be screened for HIV [1].

In general, immunocompetent individuals with HSV proctitis will follow a self-limiting course [1]. The Centers for Disease Control and Prevention (CDC) recommend antiviral treatment if HSV infection is suspected or confirmed in acute proctitis [4]. This is consistent with evidence from clinical trials, including a randomized, double-blind 1988 study that showed treatment with acyclovir 400 milligrams (mg) orally 5 times daily reduced the duration of rectal lesions and viral shedding [13]. For the first clinical episode, 7–10-day treatment with either acyclovir 400 mg orally 3 times daily, acyclovir 200 mg orally 5 times daily, valacyclovir 1 g orally twice daily, or famciclovir 250 mg orally 3 times daily should be utilized [4]. Supportive care includes measures such as pain control and warm soaks [2]. Though rectal and anogenital HSV can recur, recurrent episodes generally follow a course that is milder in symptomology and diminish in frequency over time [1, 4]. Daily suppressive antiviral therapy to prevent further episodes of herpes proctitis is not routinely recommended, but it should be considered in immunosuppressed individuals or individuals with frequent or severe recurrences [1, 4].

## 3. Conclusion

In addition to evaluation for IBD, sexually active patients presenting with proctitis should be evaluated for infectious causes such as HSV,* N. gonorrhoeae*,* C. trachomatis*, and* T. pallidum*. Empiric therapy for sexually transmitted diseases should be considered based on the clinical presentation and presence of risk factors. HIV testing should be conducted. Referral to gastroenterology for anoscopy, sigmoidoscopy, or colonoscopy is recommended. MSM, unprotected receptive anal sex, immunocompromised status, and/or positive HIV status are risk factors for HSV proctitis. External HSV lesions are absent in the majority of cases, and the absence of these lesions should not diminish suspicion for HSV infection. Although HSV proctitis will typically have a self-limited course, treatment with acyclovir or related antivirals can help reduce both the duration of rectal lesions and the duration of viral shedding.

## References

[1] Hoentjen F., Rubin D. T. (2012). Infectious proctitis: when to suspect it is not inflammatory bowel disease. *Digestive Diseases and Sciences*.

[2] Cone M. M., Whitlow C. B. (2013). Sexually transmitted and anorectal infectious diseases. *Gastroenterology Clinics of North America*.

[3] Lamb C. A., Lamb E. I., Mansfield J. C., Sankar K. N. (2013). Sexually transmitted infections manifesting as proctitis. *Frontline Gastroenterology*.

[4] Workowski K. A., Bolan G. A., Centers for Disease Control and Prevention (2015). Sexually transmitted diseases treatment guidelines, 2015. *MMWR Recommendations and Reports*.

[5] Goodell S. E., Quinn T. C., Mkrtichian E., Schuffler M. D., Holmes K. K., Corey L. (1983). Herpes simplex virus proctitis in homosexual men. Clinical, sigmoidoscopic, and histopathological features. *New England Journal of Medicine*.

[6] Chayavichitsilp P., Buckwalter V J., Krakowski A. C., Friedlander S. F. (2009). Herpes simplex. *Pediatrics in Review*.

[7] Lavery E. A., Coyle W. J. (2008). Herpes simplex virus and the alimentary tract. *Current Gastroenterology Reports*.

[8] Bissessor M., Fairley C. K., Read T., Denham I., Bradshaw C., Chen M. (2013). The etiology of infectious proctitis in men who have sex with men differs according to HIV status. *Sexually Transmitted Diseases*.

[9] Xu F., Sternberg M. R., Kottiri B. J. (2006). Trends in herpes simplex virus type 1 and type 2 seroprevalence in the United States. *The Journal of the American Medical Association*.

[10] Klausner J. D., Kohn R., Kent C. (2004). Etiology of clinical proctitis among men who have sex with men. *Clinical Infectious Diseases*.

[11] Patel R., Green J., Clarke E. (2015). 2014 UK national guideline for the management of anogenital herpes. *International Journal of STD and AIDS*.

[12] Tabet S. R., Krone M. R., Paradise M. A., Corey L., Stamm W. E., Celum C. L. (1998). Incidence of HIV and sexually transmitted diseases (STD) in a cohort of HIV-negative men who have sex with men (MSM). *AIDS*.

[13] Rompalo A. M., Mertz G. J., Davis L. G. (1988). Oral acyclovir for treatment of first-episode herpes simplex virus proctitis. *The Journal of the American Medical Association*.

